# Water Regime Influences Bulk Soil and Rhizosphere of *Cereus jamacaru* Bacterial Communities in the Brazilian Caatinga Biome

**DOI:** 10.1371/journal.pone.0073606

**Published:** 2013-09-17

**Authors:** Vanessa Nessner Kavamura, Rodrigo Gouvêa Taketani, Milena Duarte Lançoni, Fernando Dini Andreote, Rodrigo Mendes, Itamar Soares de Melo

**Affiliations:** 1 Laboratory of Environmental Microbiology, Embrapa Environment, Jaguariúna, São Paulo, Brazil; 2 Department of Soil Science, “Luiz de Queiroz” College of Agriculture, University of São Paulo, Piracicaba, São Paulo, Brazil; Radboud University Medical Centre, NCMLS, Netherlands

## Abstract

We used the T-RFLP technique combined with Ion Torrent (PGM) sequencing of 16S rRNA and multivariate analysis to study the structure of bulk soil and rhizosphere bacterial communities of a cactus, *Cereus jamacaru,* from the Brazilian Caatinga biome, which is unique to Brazil. The availability of water shapes the rhizosphere communities, resulting in different patterns during the rainy and dry seasons. Taxonomic approaches and statistical analysis revealed that the phylum Actinobacteria strongly correlated with the dry season, while samples from the rainy season exhibited a strong correlation with the phylum Proteobacteria for rhizosphere samples and with the phyla Bacteroidetes, Firmicutes, Lentisphaerae, and Tenericutes for bulk soil samples. The STAMP software also indicated that the phylum Bacteroidetes, as well as two classes in the Proteobacteria phylum (γ and δ), were the most significant ones during the rainy season. The average abundance of the phylum Actinobacteria and the genus *Bacillus* was significantly greater during the dry season. Some significant genera found during the dry season might reflect their tolerance to the extreme conditions found in the Caatinga biome. They may also indicate the ecological function that microorganisms play in providing plants with some degree of tolerance to water stress or in assisting in their development through mechanisms of growth promotion. Alterations in microbial communities can be due to the different abilities of native microorganisms to resist and adapt to environmental changes.

## Introduction

Drought is a complex and natural phenomenon that affects several parts of the world, with negative impacts on society, the economy and the environment. Bacteria living in the soil and rhizosphere of plants in dry environments may have undergone a selective pressure in order to survive. In addition to having tolerance to the extreme conditions they are exposed to, these bacteria may confer a certain level of tolerance to plants, as well as other functions such as plant growth-promotion [1] and soil maintenance due to their function and strategy in the ecosystem [2].

As suggested in other studies [3], [4], [5], we hypothesize that the availability of water affects the structure of microbial communities, resulting in different patterns during the dry and rainy seasons in the semi-arid climate of the Brazilian biome called Caatinga.

This semi-arid climate of northeast Brazil (3–17°S to 35–45°W) is determined by rainfall index, aridity index and drought risk [6] and covers approximately 8% of the country, an area of approximately 900,000 km^2^ [7]. There are two well-defined rainy and dry seasons. Rainfall can be concentrated in December and January, March and April, or May and June (unpublished data). During the dry season, there is a predominance of hot and extremely dry weather, with temperatures up to 45°C.

This exclusive, understudied Brazilian biome, the Caatinga, harbors trees and shrubs that are highly adapted to this climate. These trees and shrubs include members of the Cactaceae family, which have developed adaptive features, such as succulent tissues for water storage and long spines to reduce water loss [8]. The species *Cereus jamacaru,* known as mandacaru, is well-distributed. This biome can be considered an extreme environment due to its high temperatures, long and irregular drought periods, low water availability and high ultraviolet radiation [9], [10]. This type of environment likely contains extremophiles that are adapted to drought and high temperatures [11], but thus far there is a lack of studies on the microbial communities associated with *C. jamacaru*. Recent work by our group reported the culturable bacteria associated with *C. jamacaru* and two other cacti also present in this biome [1].

In the present study, we evaluated the structure of bulk soil and rhizosphere bacterial communities of *C. jamacaru* during dry and rainy seasons using five different sampling points and qPCR, T-RFLP, Ion Torrent (PGM) sequencing and multivariate analyses.

## Materials and Methods

### Ethics Statement

This project was conducted with the authorization of the Institute of Environment and Renewable Natural Resources (IBAMA), process number 02001.004527/2011-90, and it did not involve endangered or protected species.

### Caatinga Sampling Sites

**Figure 1:**
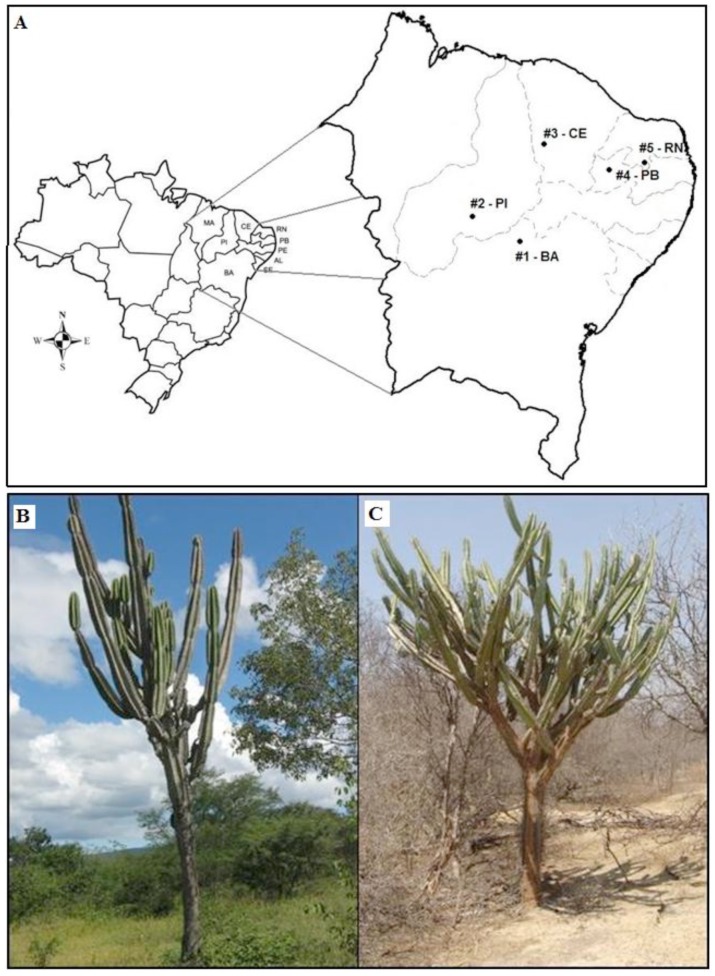
Sampling in the Caatinga biome in the semi-arid region of northeast Brazil. A - Map of Brazil in detail showing the distribution of the sampling sites comprising five states (#1 - Bahia (BA), S09°13′24.8′′; W41°05′11.4′′; #2 - Piauí (PI), S08°50′01.6′′, W42°33′13.3′′; #3 - Ceará (CE), S06°27′37.1′′, W40°44′50.5′′; #4 - Paraíba (PB), S06°42′44.2′′, W38°15′08.2′′; #5 - Rio Grande do Norte (RN), S06°39′15.6′′, W37°29′33.4′′). B and C - Pictures of *Cereus jamacaru,* a cactus found in the Caatinga biome of Brazil, during rainy (B) and dry seasons (C). (Pictures taken by the first author, 2009 and 2010).

Bulk soil (SL) and rhizosphere (RZ) samples of *Cereus jamacaru* were collected from five different sites in the Caatinga biome of semi-arid northeast Brazil (figure 1A) during two distinct seasons (rainy (RS) (figure 1B) and dry (DS) (figure 1C)). The sampling of all sites was allowed by the regulatory body concerned with the protection of wildlife and protected areas (IBAMA).

Three replicates of each bulk soil and rhizosphere sample were mixed and sub-sampled for microbial community analysis and soil chemistry characterization. The soil analysis was performed in the Center for Research and Development of Soil and Environmental Resources of the Agronomic Institute of Campinas (IAC), with macronutrient and micronutrient determination as described previously [12]. The first sampling was performed during the rainy season (May, 2009), and the second one was performed during the dry season (October, 2010). The temperature of the soil and environment ranged from 28°C to 37°C during the rainy season. During the dry season, the temperature of the soil ranged from 42°C to 50°C, and the temperature of the environment varied from 33°C to 45°C. Samples were kept in plastic bags and stored at room temperature until processing.

### DNA Extraction and Yield

Total community DNA from bulk soil and rhizosphere samples of *C. jamacaru* was extracted using a Power Soil™ DNA Isolation Kit (MOBIO Laboratories, Inc., Carlsbad, CA, USA) according to the manufacturer’s protocol. Altogether, there were forty samples: two replicates each from the five sites taken during two different seasons (dry and rainy) and taken from bulk soil or rhizosphere material. The total yield of DNA varied from 10.4 µg.µl^−1^ to 36.3 µg.µl^−1^ across all samples, with an average of 25.94±6.58 µg.µl^−1^ for bulk soil samples obtained during the rainy season, 22.26±5.62 µg.µl^−1^ for bulk soil samples obtained during the dry season, 25.35±5.25 µg.µl^−1^ for rhizosphere samples obtained during the rainy season and 23.08±7.03 µg.µl^−1^ for rhizosphere samples obtained during the dry season.

### Quantification of Total Bacterial Community by qPCR

The 16S rRNA gene was used to quantify bacterial community abundance. The quantification was performed in triplicate according to Taketani et al. 2009 [13] using an ABI Prism 7300 Cycler (Applied Biosystems). The specificity of the purified amplification products was confirmed by a melting curve analysis in which a unique peak was observed. The size of the amplicons was also checked using a 1.5% agarose gel stained with ethidium bromide. Standard curves were obtained by serially diluting a pool of bacterial 16S rRNA gene amplicons. The standard was diluted from 10^2^ to 10^9^ gene copies per microliter. The primer sets selected were P1 (5′-CCT ACG GGA GGC AGC AG-3′) and P2 (5′-ATT ACC GCG GCT GCT GG-3′) [14]. All quantitative PCR (qPCR) reactions were carried out in a 10-µl PCR mixture that contained 5 µl of Platinum SYBR Green qPCR Super Mix - UDG w/ROX (Invitrogen); 0.05 µl of each primer, 3.9 µl of Milli-Q water and 1 µl (approximately 30 ng) of environmental DNA. The PCR reactions were carried out using cycling times of 5 min at 94°C, followed by 30 cycles of 94°C for 30 sec, 57°C for 45 sec as the annealing temperature, and 72°C for 1 min. Only samples with specific melting curves and the expected amplicon sizes, as analyzed by agarose gel electrophoresis, were used for the quantification of the target group. The results were compared by Analysis of Variance and the Tukey’s test at a significance level of 5%.

### Bacterial Community Structure Analysis by T-RFLP

Amplification of 16S rRNA gene was performed with the primers 1492R (5′-TAC GGY TAC CTT GTT ACG ACT-3′) and bac27F (5′-AGA GTT TGA TCC TGG CTC AG – FAM-3′) in the following reaction: 2 µl of Dream Taq buffer, 1.2 µl of MgCl_2_, 1.6 µl of dNTP (2.5 mM), 0.07 µl of each primer (5 ρmol), 0.2 µl of Dream Taq, 2 µl of metagenomic DNA, and ultrapure sterilized water (Milli-Q) to a final volume of 20 µl. Amplification was evaluated by electrophoresis in agarose gels 1% (w/v). The amplification products were cleaved with the restriction enzyme *Hha*I (Fermentas, Life), according to the manufacturer’s instructions. Reactions were performed using a thermal cycler (Applied Biosystems) at 37°C for 90 min, followed by an increase in temperature to 65°C for 30 sec. After the restriction reaction, the products were precipitated by the addition of 2 µl of EDTA (125 mM), 2 µl of sodium acetate (3 M) and 50 µl of ethanol (100%) according to the method suggested by the manual of BigDye® Terminator v3.1 Cycle Sequencing Kit. Samples were stored at −20°C until they were analyzed.

Terminal Restriction Fragments (T-RFs) analysis was performed using ABI PRISM 3500 Genetic Analyzer (Applied Biosystems). Data obtained from sequencing were analyzed with Gene Mapper v.4.1 (Applied Biosystems) software. For T-RFs, a limit baseline of 50 fluorescence units was used to discriminate “true” peaks from the background noise in this technique. We examined T-RFs that were >50 bp and <800 bp. Peak heights were transformed to relative data (percentage of detection) [15]. T-RFLP profiles were compared among different samples using T-RF relative abundance (>1%), in which each T-RF was considered to be one Operational Taxonomic Unit (OTU). Analysis of Similarity (ANOSIM) was performed with Bray-Curtis, offering a value ranging from −1 to 1. Values close to 0 indicate no differences among groups, while values close to 1 indicate a distinction among groups [16]. Non-metric Multidimensional Scaling (NMDS) graphs indicate the relative similarity of samples through distance ordering, in which similar samples are found to be very close [16]. Analyses were performed using Past 2.12 [17] software.

### Bacterial Community Analysis using Ion Torrent (PGM) Sequencing

Each sample of DNA was amplified using the primers 967F (5′-CAA CGC GAA GAA CCT TAC C-3′) e 1046R (5′-CGA CAG CCA TGC ANC ACC T-3′) for the V6 region of 16S rRNA gene [18]; however, a different primer, 967F, with an additional tag of five nucleotides was synthesized for each sample (http://vamps.mbl.edu/). PCR reactions were carried out according to Sogin et al. [18]. After the amplification, each reaction was purified using Agencourt® AMPure® XP Reagent according to the manual provided by the manufacturer (Ion Amplicon Library Preparation (Purify the amplicon libraries), Life Technologies). At the beginning of the amplification process, we had forty samples; after the purification step, we ended with twenty samples because the replicates were joined. Each purified amplicon library was quantified using NanoDrop (Thermo Scientific), and the concentration of each sample was adjusted to prepare an equimolar pool (26 ρM); 18 µL of this pool was used for emulsion PCR according to the Ion PGM™ 200 Xpress™ Template Kit manual from Life Technologies. After the recovery of ion spheres and enrichment, samples were prepared to be loaded on a 316 chip for posterior sequencing (Ion Sequencing Kit User Guide v2.0, Life Technologies) using the Ion Personal Genome Machine™ (PGM™) (Ion Torrent, Life Technologies).

### Sequence Analyses

The initial handling of the sequences was performed using the online platform Galaxy (https://main.g2.bx.psu.edu/root), in which the raw data obtained from the sequencer Ion Personal Genome Machine™ (PGM™) (Ion Torrent, Life Technologies) were converted to FASTQ format using FASTQ Groomer [19] and were filtered by quality (95% of bases with Q20); in addition, reverse primers and adapters were removed on FASTX-toolkit. The classification of sequences was performed with mothur [20] following the recommendations of the SOP tutorial (http://www.mothur.org/wiki/Schloss_SOP) with adaptations from the Sogin tutorial (http://www.mothur.org/wiki/Sogin_data_analysis) to adjust to the short read from the V6 region. The removal of sequences (barcodes and forward primers) and library separation were performed using the trim.seqs command (settings, maxambig = 0, maxhomop = 6, bdiffs = 1, pdiffs = 2, minlength = 56, keepfirst = 60). Afterwards, the number of sequences was optimized using the unique.seqs command. Sequences were then aligned against the mothur version of the greengenes alignment by the align.seqs command using needleman as the alignment method (settings, ksize = 9 and gapopen = −1). The resulting alignment was passed through the screen.seqs command (settings, start = 4655, optimize = end, criteria = 95) to remove sequences that were outside of the desired range (i.e., the V6 region). The resulting alignment was then filtered using the filter.seqs command to remove any columns in the alignment that did not contain data. The number of sequences was once again optimized using the unique.seqs command followed by the pre.cluster command (settings, diffs = 2). The optimized alignment was used to check for chimeras using the chimera.uchime command. The resulting sequences were classified by Greengenes taxonomy using the classify.seqs command (cutoff = 60). The dist.seqs command was then used to generate a distance matrix (default settings). The sequences were then clustered into OTUs with the cluster command (average neighbor method). Richness estimators and diversity indexes were obtained by the summary.single command. The files *.groups, *.taxomony and *.names were exported to STAMP (Statistical Analysis of Metagenomic Profiles) [21] for further analysis. The comparison among multiple groups was performed using the software STAMP, which performs a statistical analysis of the sequences obtained in the samples, indicating the most abundant groups statistically and showing relevant differences in communities. Multivariate analyses were performed in Canoco 4.5 [22].

### Sequence Accession Number

The 16S rRNA reads are available at: https://main.g2.bx.psu.edu/u/vannessner/h/16s-rrna-libraries-brazilian-caatinga-biome.

## Results and Discussion

### Soil Chemistry Characteristics

All soil samples were found to be acidic, with site #2 presenting the lowest pH (table S1). Most soil samples from the five sampling sites presented significantly different values (according to Tukey’s test at 5%) for most of the measured parameters. There is a high variability among the sampling sites, as observed in the clustering analysis using the unweighted pair-group average (UPGMA) with Euclidean similarity measure (figure S1), in which no pattern was found across different locations or seasons.

### Abundance of Bacterial Communities by Quantitative PCR

**Figure 2:**
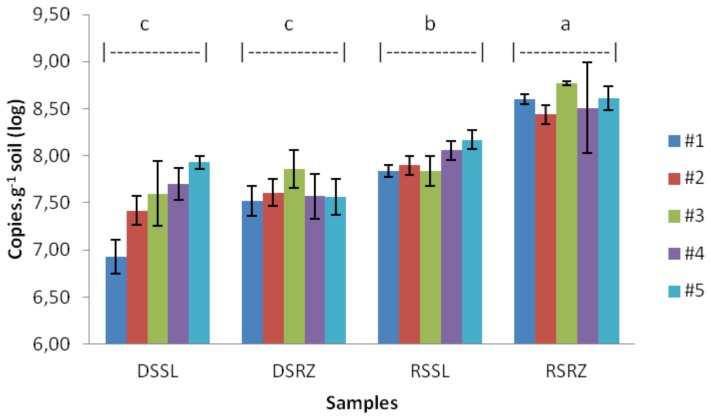
Quantification by qPCR of bacterial 16S rRNA gene copies in samples obtained during the rainy (RS) and dry (DS) seasons for both bulk soil (SL) and rhizosphere (RZ). Values indicate the average of three replications, and bars represent standard errors.

The number of bacterial 16S rRNA gene copies identified in samples shows a general trend of increased abundance of bacteria in samples obtained during the rainy season, as compared to samples obtained during the dry season. Tukey’s test confirms this result, with rhizosphere samples during the rainy season (RSRZ) being significantly more abundant than others (figure 2).

Samples from the rhizosphere during the rainy season have higher numbers of copies of the 16S rRNA gene compared to all other samples (p<0.01). In all sampling sites for the dry season, the rhizosphere (DSRZ) sample values ranged from 7.52 to 7.86 log of 16S rRNA gene copies per gram of soil. For rhizosphere samples obtained during the rainy season, the values are higher, ranging from 8.44 to 8.77 log of gene copies per gram of soil. Samples obtained from bulk soil ranged from 6.93 to 7.93 log for the dry season (DSSL) and from 7.84 to 8.17 log for the rainy season (RSSL). The transition from the rainy season to the dry season caused a variation in the rhizosphere samples, with a decrease of 9.8 to 12.6% in the numbers of copies per gram of soil. In the bulk soil samples, the decrease ranged between 2.9 and 11.6%, a more marked variation.

### Bacterial Community Patterns

After observing a higher abundance of bacteria during the rainy season, we used T-RFLP to test whether the structure of the bacterial community varied. Non-metric multidimensional scaling (NMDS) was used with ANOSIM. The spatial variation between the five different sampling points was not significant, yielding a value of R close to zero (R = −0.002, p = 0.456). There is no clear division between the sampling sites (figure S2A).

The variation between bulk soil and rhizosphere samples produced a very low value of R (R = 0.054, p<0.05). There is a clearer separation between samples obtained during the rainy season (figure S2B), whereas bulk soil and rhizosphere samples obtained during the dry season slightly mix with each other (figure S2C), indicating a greater similarity between them. The selective pressure exerted by the dry season eliminated the rhizosphere effect, thus forming a tighter cluster.

**Figure 3:**
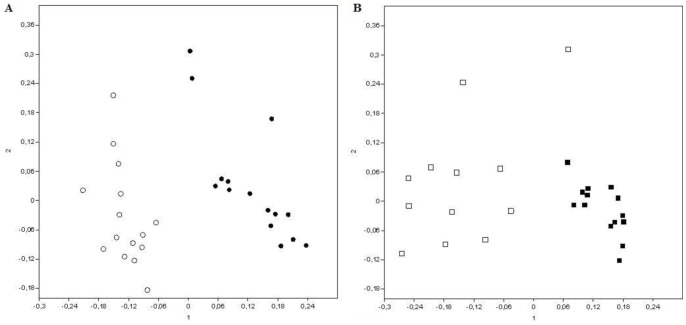
Non-metric multidimensional scaling (NMDS) of bacterial communities determined by T-RFLP, showing the seasonal variation among bulk soil (A) and rhizosphere (B) samples. Bulk soil samples are represented by circles and rhizosphere samples are represented by squares. Rainy season samples are represented by the white color and dry season samples are represented by the black color.

Figure 3 shows a significant seasonal variation, with R = 0.626 (p<0.001). There is a clear separation between the rainy and dry seasons, both for bulk soil (figure 3A) and rhizosphere samples (figure 3B). Thus, despite the high variability found across the sampling sites, the distinction between seasons is evident.

These results suggest a change in the structure of the bacterial community according to seasonal variation (rainy vs. dry), as reported by several authors [5], [23], [24], [25], [26]. Several factors contribute to the design of the composition of bacterial communities [27]. In this analysis, it is evident that the community structure is shaped primarily by the presence or absence of water. The same result has been found for the rhizosphere of two leguminous trees (*Mimosa tenuiflora* and *Piptadenia stipulacea*) growing in the same Caatinga biome [28].

### Bacterial Diversity Based on 16S rRNA Sequencing

We used a high-resolution technique to determine which groups were present in each season. A total of 590,043 sequences was obtained by Ion Torrent (PGM) Sequencing. Mothur classified 127,348 sequences as belonging to the domain Bacteria, of which 55.33% were grouped into thirty phyla and the remaining 44.67% remained unclassified. Twelve phyla showed relative frequencies greater than 1% for most samples.

Clustering analysis using Ward’s method clearly distinguishes samples obtained during the different seasons, corroborating previous observations (figure S3). Samples obtained during the dry season form one group, while samples obtained during the rainy season form two distinct groups, separating the rhizosphere samples from the bulk soil samples.

**Figure 4:**
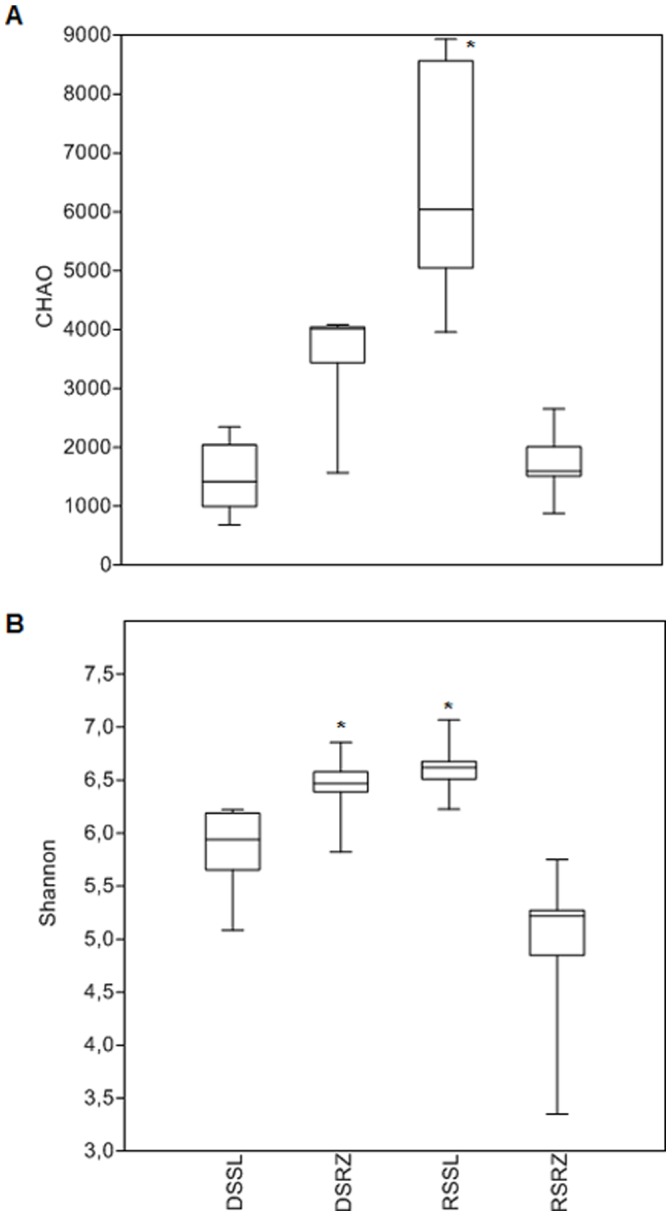
Estimation of total species richness obtained by the Chao index (A) and diversity using the Shannon-Wiener index (B) for samples from bulk soil during the dry season (DSSL), rhizosphere during the dry season (DSRZ), bulk soil during the rainy season (RSSL) and rhizosphere during the rainy season (RSRZ). Significant values according to Tukey’s test (p<0.01) are indicated by an *.

The total species richness estimated by the Chao index was significantly higher (p<0.01) for bulk soil samples obtained during the rainy season (figure 4A), and diversity was also significantly higher (p<0.01) for rhizosphere samples obtained during the dry season and for bulk soil samples obtained during the rainy season (figure 4B).

**Figure 5:**
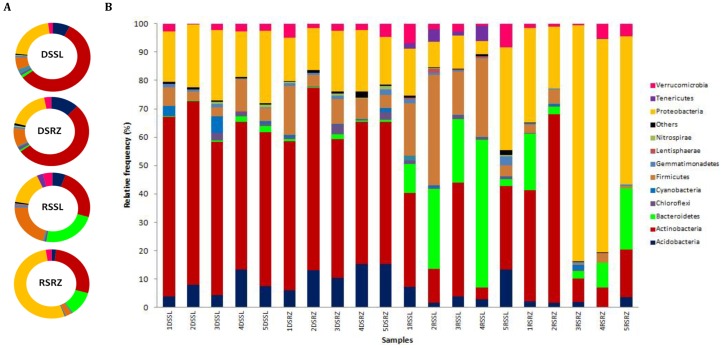
Classified sequences divided by phyla with relative frequencies higher than 1% (for the majority of the samples). A - Distribution of phyla, divided by samples. B - Samples obtained for the five different sampling sites from bulk soil during the dry season (DSSL), rhizosphere during the dry season (DSRZ), bulk soil during the rainy season (RSSL) and rhizosphere during the rainy season (RSRZ). “Others” include eighteen phyla with relative frequencies lower than 1%: AD3, Armatinonadetes, Candidatus Poribacteria, CCM11b, Chlamydiae, Elusimicrobia, Fibrobacteres, Fusobacteria, NC10, Planctomycetes, SC3, SC4, SPAM, Spirochaetes, Synergistetes, TM7, WPS-2 and WS3.

In general, Actinobacteria was the dominant phylum, especially for samples obtained during the dry season (figure 5A). This phylum was also found in great abundance in rhizosphere samples obtained from a Mexican semi-arid cactus [29]. Bacteroidetes and Proteobacteria were detected with higher frequency during the rainy season, with Proteobacteria being more frequent in rhizosphere samples (figure 5B). The number of sequences affiliated with the phylum Acidobacteria was also higher in samples obtained during the dry season, comprising 7% and 12% of the reads from the bulk soil and the rhizosphere, respectively. Although being quite abundant in soil samples [30] and also in semi-arid environments [31], this phylum was not detected with high frequency in this study.

**Figure 6:**
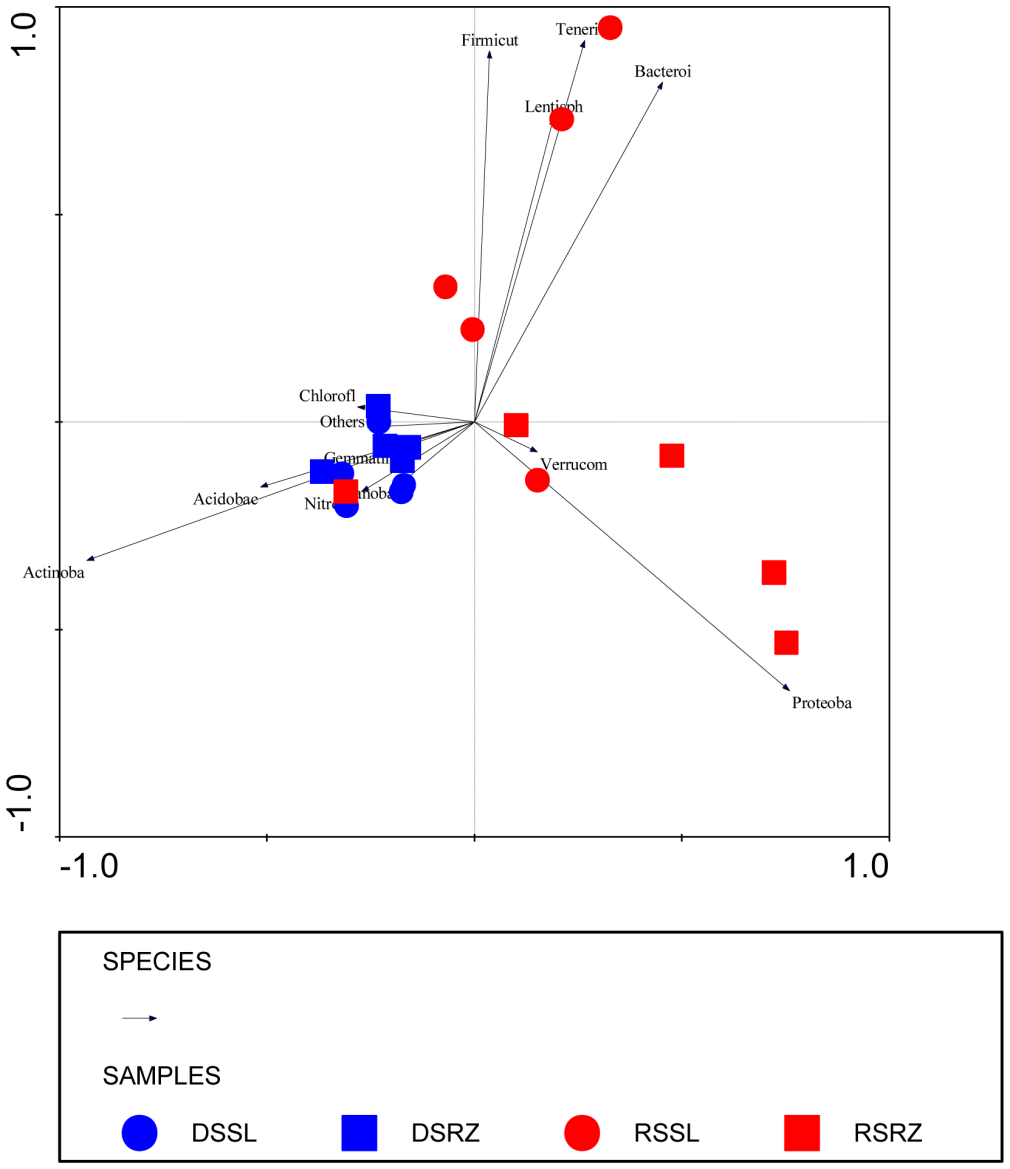
PCA of the 16S libraries based on taxonomic affiliation of reads determined by mothur. Samples are bulk soil during the rainy season (RSSL), bulk soil during the dry season (DSSL), rhizosphere during the rainy season (RSRZ) and rhizosphere during the dry season (DSRZ). The most common phyla are: Acidobacteria (Acidobac), Actinobacteria (Actinoba), Bacteroidetes (Bacteroi), Chloroflexi, Cyanobacteria (Cyanoba), Firmicutes (Firmicut), Gemmatimonadetes (Gemmati), Lentisphaerae (Lentisph), Nitrospirae (Nitro), Others, Proteobacteria (Proteoba), Tenericutes (Tener) and Verrucomicrobia (Verrucom).

Principal Component Analysis (PCA) based on the relative frequencies of different phyla confirmed the results obtained from T-RFLP analysis, showing a clear separation between samples obtained from different seasons (figure 6). Samples obtained during the rainy season are more different from each other than samples obtained during the dry season. Based on the size of the arrows, samples from the dry season showed a stronger correlation to the phylum Actinobacteria and slightly correlated with the phylum Acidobacteria. The other phyla of Bacteroidetes, Lentisphaerae, Firmicutes, Proteobacteria and Tenericutes correlated with samples obtained during the rainy season, with rhizosphere samples correlating strongly with Proteobacteria, and the other four phyla correlating with bulk soil samples. To assess these differences statistically, comparisons were made among multiple groups using the software STAMP based on the criterion of the season. Significant differences (p<0.05) in the percentage of sequences were observed for some phyla. The numbers of representatives of Acidobacteria, Actinobacteria, and Nitrospirae were higher in the dry season; Bacteroidetes and Tenericutes (p = 0.05) had the largest representation during the rainy season. These data corroborate the data of two other works that reported that the abundance of representatives of the phylum Actinobacteria tends to be lower in damper soils [32], [33]. Actinobacteria were found to increase in abundance during an experiment of throughfall exclusion [34]. Correspondingly, Proteobacteria and Bacteroidetes showed increased abundance during the rainy season, along with a drop in the proportion of Actinobacteria during the same period [35].

These differences might be due to the strategies adopted by these microorganisms, in which abundant groups during drought have a strategy of slow growth as oligotrophs, while favored groups during the rainy season might have quick responses to high resource availability, indicating a copiotrophic group [2].

### Influence of Season in the Distribution of Microorganisms and their Possible Role in the Environment

**Figure 7:**
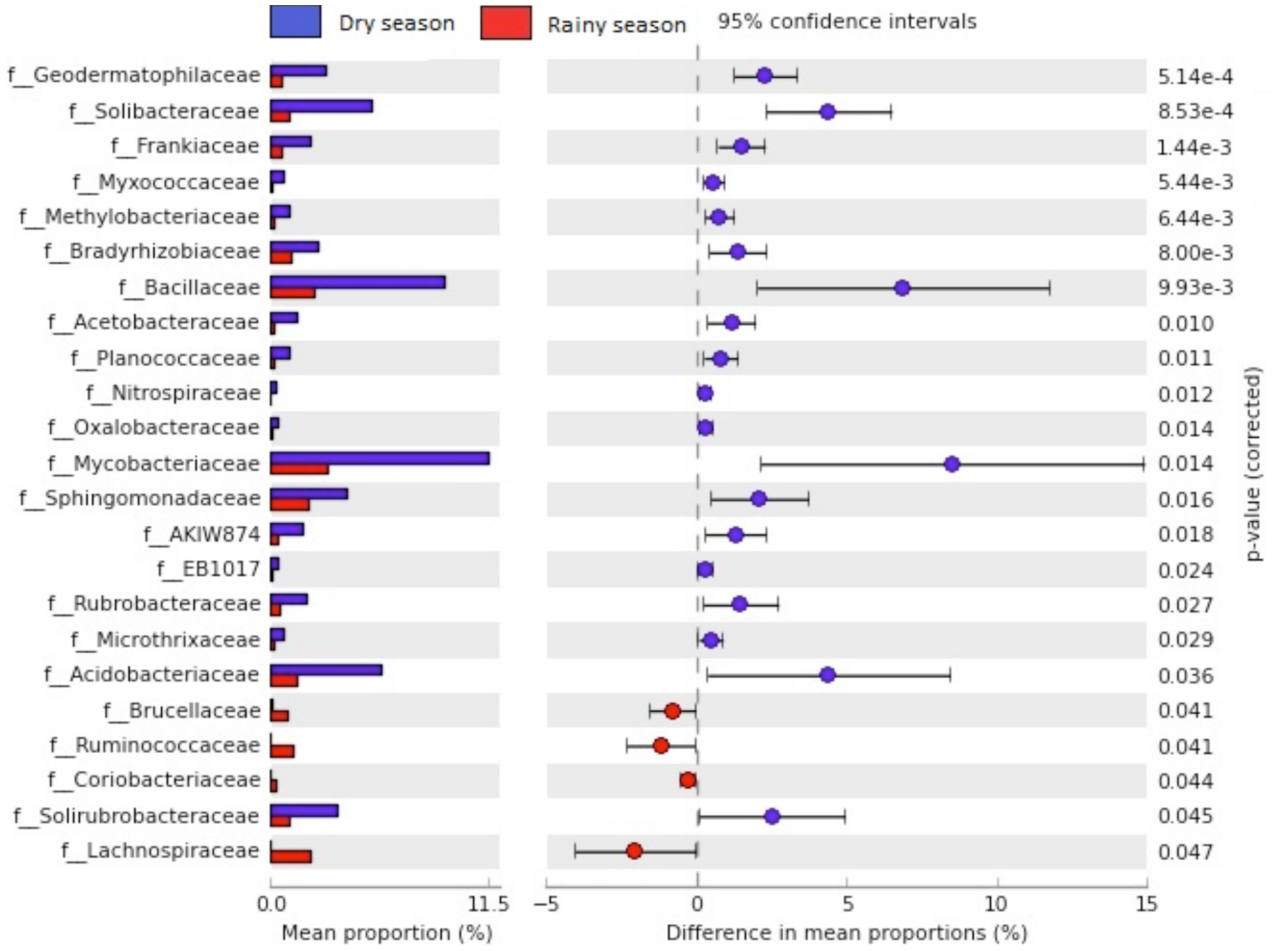
The twenty-three most significant families (p<0.05) with respect to the season.

At a finer phylogenetic resolution (that is, family and genus level), twenty-one families were significantly more abundant (p<0.05) during the dry season and twelve were significantly more abundant during the rainy season. Figure 7 shows the twenty-three most significant families. The Bacillaceae family, belonging to the phylum Firmicutes, class Bacilli, and order Bacillales, comprises Gram-positive endospore-forming microorganisms. The endospores are specialized resistance structures that allow the survival of microorganisms for extended periods in dry soils [36] and also give protection against several environmental stressors [37]. In a study conducted on the rhizosphere of the cactus *Mammillaria carnea* during rainy and dry seasons, the phylum Firmicutes had increased its abundance during the dry period due primarily to the class Clostridia [5], which also has endospore-forming bacteria [38].

**Figure 8:**
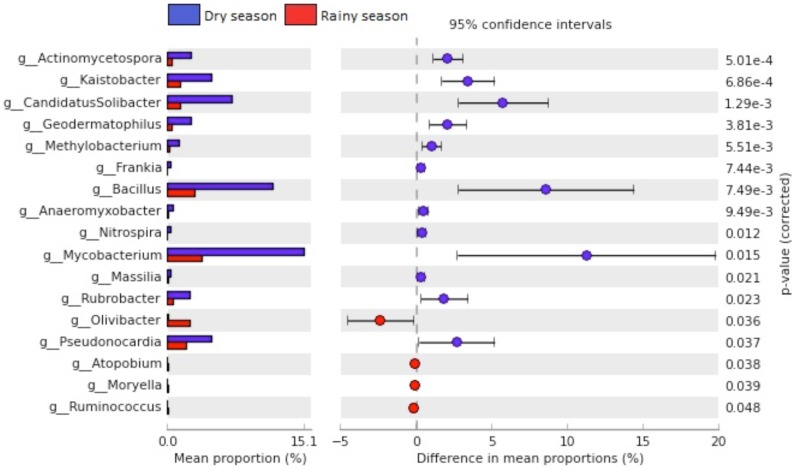
The seventeen most significant genera (p<0.05) with respect to the season.

At the genus level, figure 8 shows the seventeen most significant genera, with thirteen more frequent during the dry season and four more frequent during the rainy season. The significant presence of one genus in one season over another does not exclude it from the other season, and vice-versa. For example, Bacilli that were identified at a significantly greater proportion during the dry season are also present during the rainy season but are enriched under the dry season. Because the sampling season was the major factor for differences in genera, the most representative genera might have developed mechanisms of adaptation to the unfavorable conditions imposed by water stress and other factors. Such adaptations might involve tolerance to high temperatures [39], desiccation tolerance genes [40], pigment production for protection against UV radiation [41], production of thermostable enzymes [42] and production of intracellular osmolytes [43]. We believe that the presence of a large number of sequences belonging to the genus *Bacillus* can be explained by the fact that endospore-forming bacteria might be favored in these types of environments due to resistance to heat and desiccation [44]. Six out of thirteen genera belong to the phylum Actinobacteria. They are also able to withstand harsh environments by producing spores resistant to desiccation and extreme heat [45]. Unlike the *Bacillus* genus, Gram-positive members with a high G+C content (Actinobacteria) are not capable of forming endospores. Some form spores similar to those produced by the genus *Streptomyces*, which are different from endospores and serve as a specialized reproductive structure that germinates under favorable conditions [46]. When spore production is not observed, they enter a dormant state similar to *Mycobacterium* characterized by low metabolic activity [47].

It is known that different plant species host specific microbial communities because plants are able to shape their rhizosphere microbiome [48], [49]. The higher detection of some genera exclusively in the dry season can be due to the ecological role that some microorganisms play in the environment, such as providing a degree of tolerance against drought stress for plants they associate with [50]. Drought tolerance may come from the improvement of soil physical properties, such as soil aggregation through the production of exopolysaccharides and biofilm or protection by the production of osmolytes [51], [52]. More details on how microorganisms help plants tolerate abiotic stress, i.e., drought, and even promote plant growth are provided by Kavamura et al. [1].

## Conclusion and Future Perspectives

In general, qPCR and T-RFLP analysis combined with Ion Torrent (PGM) sequencing allowed us to confirm that the bulk soil and rhizosphere bacterial community of *C. jamacaru* showed a clear alteration between the rainy and dry seasons.

During the rainy season, a higher proportion of Gram-negative bacteria, especially those represented by Bacteroidetes and some classes of Proteobacteria, were observed. These include several microorganisms in soil that are important not only for nutrient cycling [53] but also for the whole ecosystem [54]. In the dry season, a higher proportion of Gram-positive bacteria, represented by the phylum Actinobacteria and the genus *Bacillus,* was observed.

We believe that the presence of dominant groups during the dry season, which are also present to a lesser extent during the rainy season, suggests that in soil, there are drought tolerant microorganisms. When conditions become unfavorable, sensitive groups decrease in proportion and the tolerant groups stand out due to the above-mentioned tolerance mechanisms. When the rainy season comes, drought-sensitive microorganisms are capable of growing rapidly, and they reestablish the community [55].

Changes in microbial communities can be observed due to the different abilities of native microorganisms to resist and adapt to environmental changes. However, these skills require a lot of energy and vary according to each microorganism [56]. Microorganisms fall into four categories depending on their ability to resist or adapt to environmental changes: i) microorganisms that have no mechanism for acclimatization; ii) microorganisms that only have acclimatization mechanisms; iii) microorganisms that possess inherent resistance; and iv) microorganisms with inherent resistance plus acclimatization mechanisms [57].

Gram-negative bacteria tend to fall into category ii, and Gram-positive bacteria tend to fall into categories iii or iv.

Thus, Gram-positive bacteria can be more resistant to rain/drought events, which is confirmed in this study. This result highlights a certain level of selection of microorganisms with effective tolerance mechanisms because bacterial communities that regularly suffer from stress episodes appear to be more tolerant than those that suffer from these episodes sporadically.

This work represents the first effort to better understand the bacteria associated with a widely distributed cactus in the Caatinga semi-arid biome of Brazil. Our group is now focusing on a complete description of the metagenome of this environment describing the metabolic pathways of the microorganisms inhabiting this biome to enable further understanding of how microbial communities remain active during the dry season, how they recover and the dynamics underlying these changes.

## Supporting Information

Figure S1 **Clustering analysis of sampling sites, using UPGMA with Euclidian distance.** Comparison during the rainy season (RS) and dry season (DS) for bulk soil samples (SL) for the five different sampling points: #1, #2, #3, #4 and #5.
(TIF)

Figure S2 **Non-metric multidimensional scaling (NMDS) of bacterial communities determined by T-RFLP, showing the spatial variation (A) and source variation (B and C).** Bulk soil samples are represented by circles and rhizosphere samples are represented by squares. Rainy season is represented by the white color and dry season is represented by the black color. The five different sampling points are represented by a cross (#1), a white triangle (#2), an x (#3), a black triangle (#4), and a rectangle (#5).
(TIF)

Figure S3 **Clustering analysis using Ward’s method obtained from 16S rRNA sequences showing a clear division according to the season.** Dry season samples (DS) form one cluster, while rainy season samples (RS) form two separate clusters, one including bulk soil samples (SL) and the other including rhizosphere samples (RZ).
(TIF)

Table S1 Soil chemical features of each site for both seasons. Values are presented as the average ± standard errors (n = 3). In each line, values followed by the same letters do not differ statistically according to Tukey’s test at 5%.
(DOCX)
