# Supplementary figures and images for: Water Regime Influences Bulk Soil and Rhizosphere of Cereus jamacaru Bacterial Communities in the Brazilian Caatinga Biome
Source: PLoS One. 2013 Sep 17;8(9):e73606. doi: 10.1371/journal.pone.0073606 (PMC3775785; doi:10.1371/journal.pone.0073606)

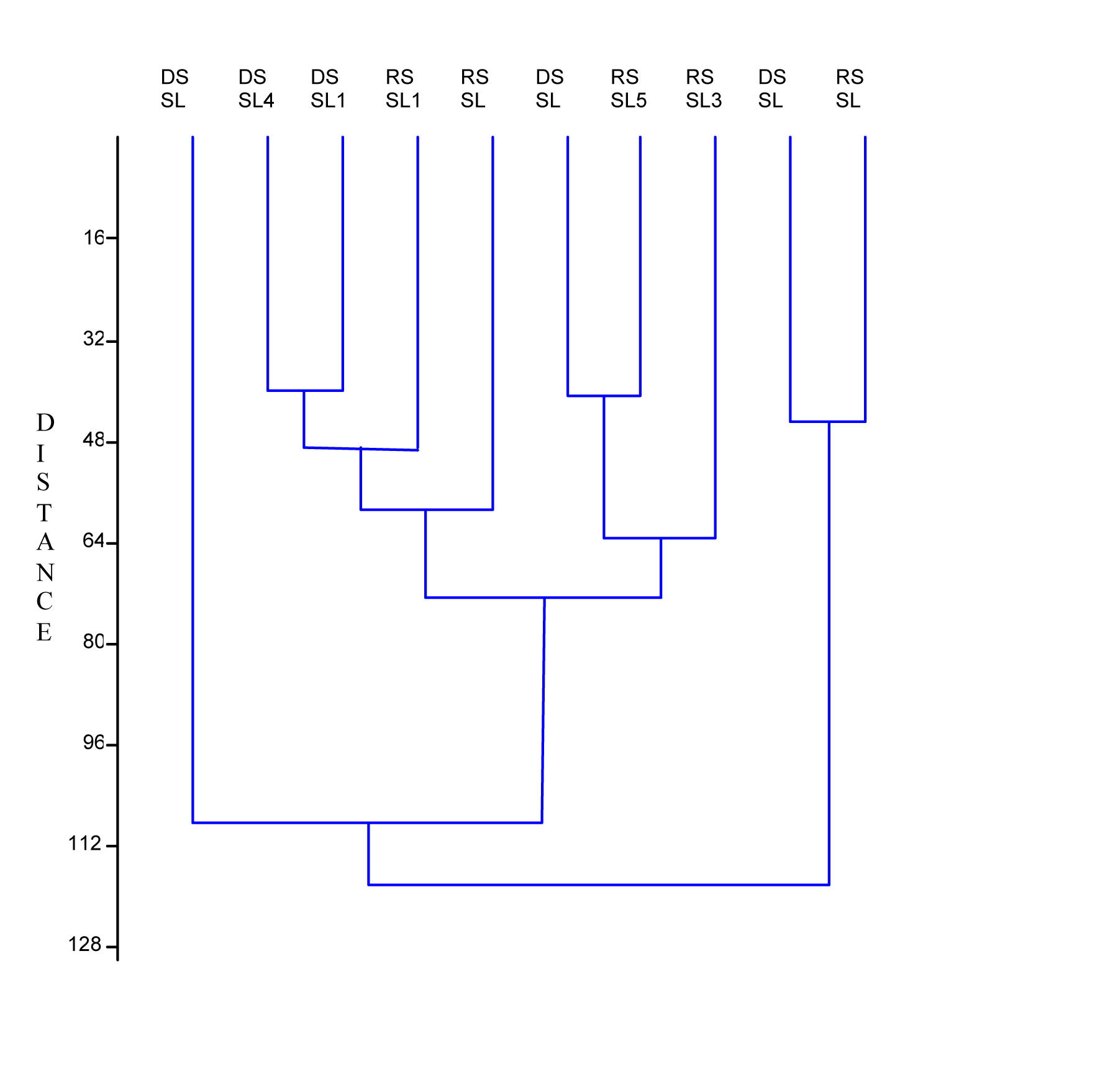

Supplement: Figure S1 — Clustering analysis of sampling sites, using UPGMA with Euclidian distance. Comparison during the rainy season (RS) and dry season (DS) for bulk soil samples (SL) for the five different sampling points: #1, #2, #3, #4 and #5. (TIF) [file pone.0073606.s001.tif]

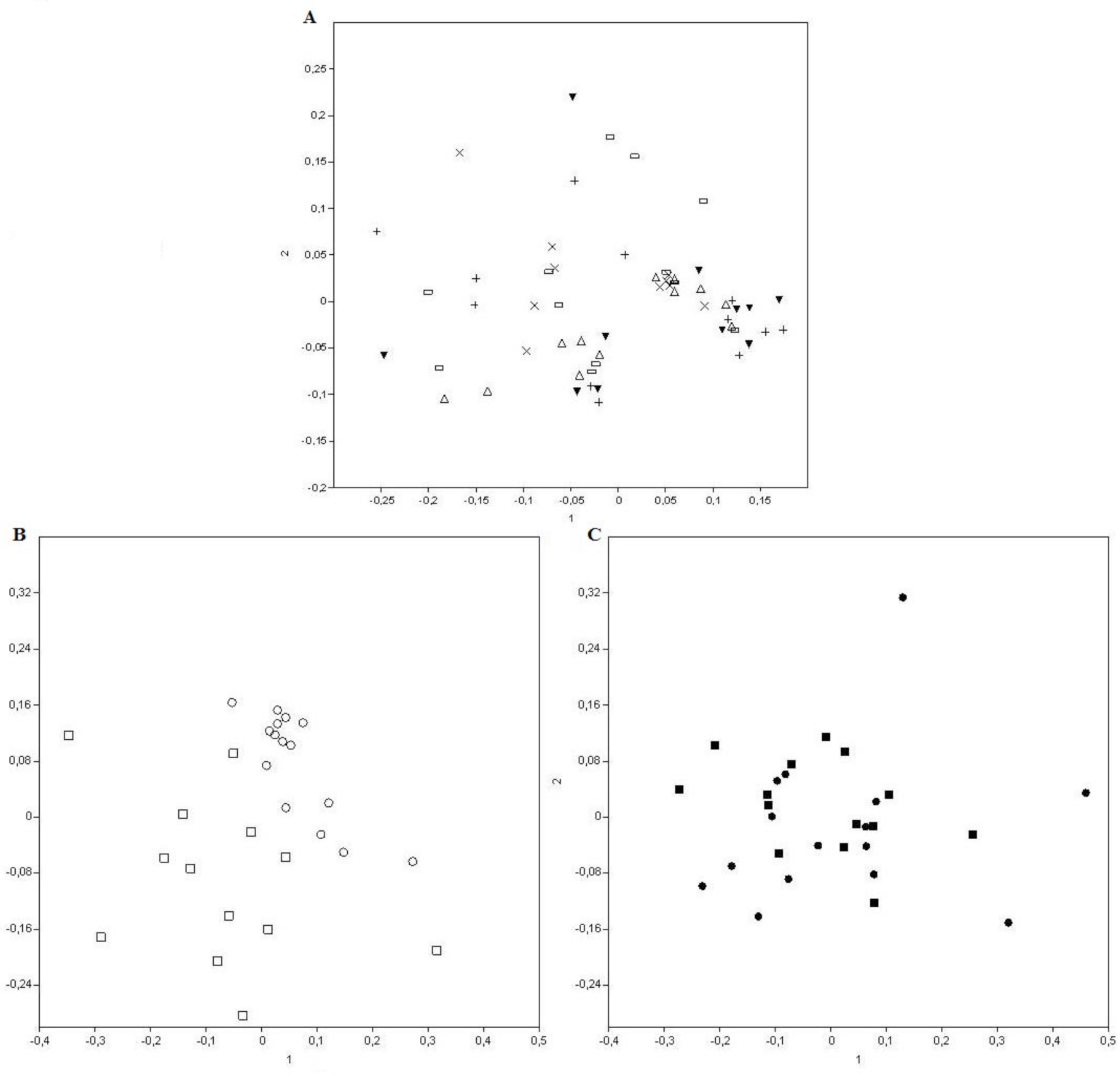

Supplement: Figure S2 — Non-metric multidimensional scaling (NMDS) of bacterial communities determined by T-RFLP, showing the spatial variation (A) and source variation (B and C). Bulk soil samples are represented by circles and rhizosphere samples are represented by squares. Rainy season is represented by the white color and dry season is represented by the black color. The five different sampling points are represented by a cross (#1), a white triangle (#2), an x (#3), a black triangle (#4), and a rectangle (#5). (TIF) [file pone.0073606.s002.tif]

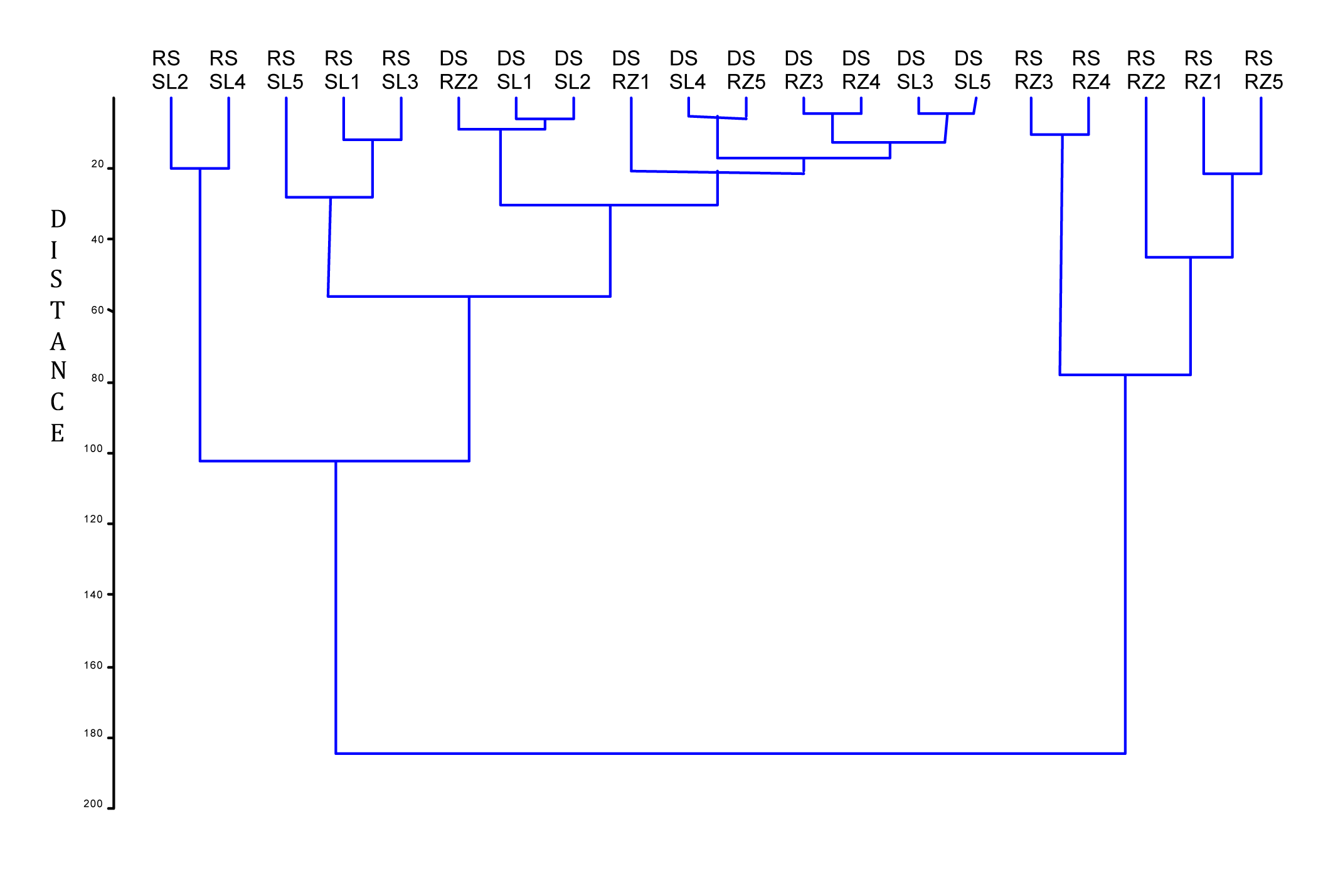

Supplement: Figure S3 — Clustering analysis using Ward’s method obtained from 16S rRNA sequences showing a clear division according to the season. Dry season samples (DS) form one cluster, while rainy season samples (RS) form two separate clusters, one including bulk soil samples (SL) and the other including rhizosphere samples (RZ). (TIF) [file pone.0073606.s003.tif]
