# Supplementary material for: Water Regime Influences Bulk Soil and Rhizosphere of Cereus jamacaru Bacterial Communities in the Brazilian Caatinga Biome
Source: PLoS One. 2013 Sep 17;8(9):e73606. doi: 10.1371/journal.pone.0073606 (PMC3775785; doi:10.1371/journal.pone.0073606)
Supplement: Table S1 — Soil chemical features of each site for both seasons. Values are presented as the average ± standard errors (n = 3). In each line, values followed by the same letters do not differ statistically according to Tukey’s test at 5%. (DOCX) [file pone.0073606.s004.docx]

|  | **Rainy season** | | | | | **Dry season** | | | | |
| --- | --- | --- | --- | --- | --- | --- | --- | --- | --- | --- |
| **Sites** | #1 | #2 | #3 | #4 | #5 | #1 | #2 | #3 | #4 | #5 |
| **OM** | 24.50±0.29^de^ | 20.50±0.29^f^ | 24.00±0.00^de^ | 24.50±0.29^de^ | 37.00±0.00^b^ | 23.00±0.00^def^ | 22.00±0.00^ef^ | 43.00±1.73^a^ | 33.50±0.29^c^ | 25.50±0.29^d^ |
| **pH** | 5.80±0.00^c^ | 4.15±0.03^f^ | 6.20±0.00^b^ | 5.25±0.03^e^ | 5.65±0.03^d^ | 5.60±0.00^d^ | 4.20±0.00^f^ | 5.65±0.03^d^ | 5.30±0.00^e^ | 6.30±0.00^a^ |
| **P** | 5.00±0.00^c^ | 15.00±0.58^c^ | 3.00±0.00^c^ | 3.50±0.29^c^ | 49.50±10.10^a^ | 6.00±0.00^c^ | 9.00±0.00^c^ | 15.50±0.29^bc^ | 6.50±0.29^c^ | 31.50±0.87^b^ |
| **K** | 2.55±0.03^e^ | 0.70±0.00^g^ | 1.40±0.00^f^ | 3.60±0.12^d^ | 6.40±0.23^b^ | 2.30±0.06^e^ | 1.50±0.00^f^ | 4.60±0.29^c^ | 8.25±0.03^a^ | 4.65±0.03^c^ |
| **Ca** | 31.50±0.29^de^ | 4.50±0.29^g^ | 39.00±0.00^cd^ | 19.50±1.44^f^ | 50.50±3.18^b^ | 23.00±0.58^ef^ | 8.50±0.29^g^ | 64.00±4.04^a^ | 23.50±0.29^ef^ | 46.50±0.29^bc^ |
| **Mg** | 6.00±0.00^e^ | 1.00±0.00^f^ | 24.00±0.00^b^ | 6.00±0.58^e^ | 12.00±0.58^c^ | 5.00±0.00^e^ | 1.50±0.29^f^ | 37.50±0.29^a^ | 8.00±0.00^d^ | 13.00±0.00^c^ |
| **H+Al** | 17.00±0.58^fg^ | 32.50±0.87^b^ | 15.00±0.00^g^ | 22.00±0.00^e^ | 23.50±0.87^de^ | 19.00±0.58^f^ | 36.00±1.15^a^ | 25.00±0.00^d^ | 28.00±0.00^c^ | 15.00±0.00^g^ |
| **S.B.** | 39.45±0.20^c^ | 6.50±0.06^d^ | 63.90±0.00^b^ | 29.30±2.02^c^ | 69.15±4.19^b^ | 30.10±0.35^c^ | 11.45±0.20^d^ | 106.70±4.79^a^ | 39.65±0.14^c^ | 63.90±0.23^b^ |
| **CEC** | 56.75±0.32^de^ | 39.05±0.95^f^ | 78.70±0.00^c^ | 51.80±2.02^e^ | 92.90±4.91^b^ | 49.35±0.95^ef^ | 47.65±0.89^ef^ | 131.70±4.79^a^ | 67.40±0.17^cd^ | 78.65±0.26^c^ |
| **V%** | 69.50±0.87^c^ | 17.00±0.58^g^ | 81.00±0.00^a^ | 56.00±1.73^e^ | 74.00±0.58^b^ | 61.00±0.58^f^ | 24.50±0.87^f^ | 81.00±0.58^a^ | 59.00±0.00^de^ | 81.00±0.00^a^ |
| **B** | 0.32±0.00^ab^ | 0.23±0.00^de^ | 0.18±0.00^fg^ | 0.16±0.01^g^ | 0.24±0.00^de^ | 0.36±0.00^a^ | 0.35±0.00^a^ | 0.29±0.03^bc^ | 0.26±0.00^cd^ | 0.21±0.01^ef^ |
| **Cu** | 0.60±0.00^c^ | 0.25±0.03^cd^ | 1.00±0.00^b^ | 0.20±0.00^d^ | 0.50±0.06^cd^ | 0.50±0.00^cd^ | 0.30±0.00^cd^ | 2.90±0.23^a^ | 0.40±0.00^cd^ | 0.40±0.00^cd^ |
| **Fe** | 12.00±0.00^f^ | 125.00±10.97^a^ | 76.00±0.00^b^ | 67.00±0.58^bc^ | 50.00±2.31^cd^ | 16.50±0.29^f^ | 84.00±5.77^b^ | 68.00±6.35^bc^ | 44.00±2.89^de^ | 23.00±0.00^ef^ |
| **Mn** | 21.00±0.29^e^ | 3.20±0.06^g^ | 51.00±0.00^b^ | 18.55±0.66^e^ | 41.45±3.44^c^ | 65.55±1.24^a^ | 12.40±0.00^f^ | 53.40±0.17^b^ | 52.45±0.20^b^ | 32.40±0.00^d^ |
| **Zn** | 0.70±0.00^d^ | 4.85±0.66^c^ | 12.30±0.00^a^ | 3.75±0.09^c^ | 7.25±0.49^b^ | 1.05±0.03^d^ | 0.75±0.03^d^ | 1.95±0.26^d^ | 1.20±0.00^d^ | 1.05±0.03^d^ |
